# Surviving on a Rock, but for How Long? Deviations in the Thermoregulatory Strategy of the Milos Wall Lizard (*Podarcis milensis*)

**DOI:** 10.3390/ani14213087

**Published:** 2024-10-26

**Authors:** Panayiotis Pafilis, Chloe Adamopoulou, Antonis Antonopoulos, Aris Deimezis-Tsikoutas, Apostolos Christopoulos, Kostas Sagonas

**Affiliations:** 1 Section of Zoology and Marine Biology, Department of Biology, National and Kapodistrian University of Athens, Panepistimioupolis, 15772 Athens, Greece; cadam@biol.uoa.gr (C.A.); antantonop@biol.uoa.gr (A.A.); adeime@biol.uoa.gr (A.D.-T.); apochris@biol.uoa.gr (A.C.); 2 Museum of Zoology, National and Kapodistrian University of Athens, Panepistimioupolis, 15772 Athens, Greece; 3 Section of Zoology, Department of Biology, Aristotle University of Thessaloniki, Panepistimioupolis, 54643 Thessaloniki, Greece; ksagonas@bio.auth.gr

**Keywords:** ectotherms, thermoregulation, islands, lizards, conservation, global warming

## Abstract

Reptiles are unable to generate metabolic heat and regulate body temperature behaviorally depending on environmental conditions. The thermal quality of their habitat is therefore of pivotal importance for their survival. Lizards render themselves as ideal ectothermic models, and their thermal biology has been extensively studied. In this work, we focused on the thermoregulatory performance of the endemic Milos wall lizard (*Podarcis milensis*) (Milos Archipelago, Aegean Sea, Greece). Applying the same standard methodology, we estimated the effectiveness of thermoregulation (*E*) taking into account the three main thermal parameters: body (*T_b_*, the temperature of active animals in the field), environmental (*T_e_*, the temperature that animals would achieve in the field if passively conform to the environment) and preferred temperatures (*T_pref_*, the temperatures an animal achieves in a laboratory thermal gradient). Here, we compare the thermoregulatory profile of two remote rocky islet populations, Falconera and Velopoula, with the Milos Island population. We collected *T_b_* values from active lizards as well as *T_e_* from specially designed copper models, which were appropriately placed in the field so as to cover all possible microhabitats. Lizards were then transported to the laboratory where we assessed their *T_prefs_*. Falconera and Velopoula populations showed the same high thermoregulatory effectiveness as that of Milos Island (*E_Falconera_* = 0.97, *E_Velopoula_* = 0.95, *E_Milos_* = 0.89). However, when we used an alternative evaluation of the thermoregulatory strategy, the *E* values outlined a much more effective thermoregulation for the islets: *de*-*db_Falconera_* = 6.97, *de*-*db_Velopoula_* = 11.54, *de*-*db_Milos_* = 4.27. The adverse conditions on the islets outline a demanding habitat of low thermal quality that dictates effective thermoregulation. However, the trend of increasing temperatures depicts an even harsher environment for the years to come. Could lizards that have already achieved the highest thermoregulatory effectiveness and cannot escape from the isolated islets they dwell cope with these new conditions? This is the kind of questions to which conservation biology will be called upon to respond.

## Simple Summary

Insularity shapes many aspects of animal biology, and thermal physiology is not an exception. However, not all islands affect animals in the same way. Here, we aim to shed light on the thermal adaptations that animals attain on small islets of the Aegean Sea, Greece. We compare the thermoregulatory profile of the endemic Milos wall lizards (*Podarcis milensis*) of two remote islets with the Milos Island population. Our results suggest that the islet lizards achieve much more effective thermoregulation than the main island in response to the low thermal quality of these demanding habitats. However, global warming poses further challenges to these populations. Even though they are already accurate, precise and effective thermoregulators, they would have—literally—nowhere to go in the case of higher environmental temperatures.

## 1. Introduction

Islands always fascinated evolutionary biologists since Darwin’s time. These well-defined ecosystems, often hosting simplified communities with dense and tame populations, lend themselves as a natural laboratory for testing evolutionary scenarios [1]. Island life deviates in many ways from the mainland norms in response to the particular insular conditions [2]; e.g., the Island Rule [3] predicts that island conditions may impose dramatic shifts in body size; the Island Syndrome [4] anticipates that insular animals would produce fewer but larger offspring, while the so-called island naïveté [5] describes the decrease in vigilance in predator-free islands. These principles apply to insular lizards that have been largely used as models in island biology [6,7,8].

Justifiably, special emphasis has been placed on the thermal biology of island lizards [9,10,11]. All aspects of saurian biology receive the decisive impact of temperature [12,13]. Being ectothermic organisms as reptiles, lizards primarily rely on environmental temperatures to effectively thermoregulate [14,15]. Parameters such as seasonality, habitat heterogeneity, elevation, latitude and altitude influence environmental temperatures to a large extend and thus impose appropriate shifts in thermoregulation [16,17,18]. Insularity is another parameter that distinctively affects body temperature [19,20].

In the mainland, thermal conditions are often challenging, and lizards have to be quite effective thermoregulators to deal with such demanding habitats [21,22]. Although such conditions are rather common in the mainland, islands have comparatively more benign climate thanks to the buffering sea effect [1,23]. Because of the higher quality of insular habitats, lizards may afford a less effective thermoregulation and exert more limited efforts than their peers from mainland populations [19,24,25]. However, on small islets, this pattern of relaxed thermoregulation may be challenged due to characteristics associated with the limited area. Small islets have low habitat heterogeneity and usually low elevation and thus are exposed to high winds [26,27]. To compensate for these small island effects, lizards have to achieve high thermoregulation effectiveness [28,29,30]. In sum, islet lizards may shift their thermal biology to deal with the particular conditions prevailing therein [31,32].

Lizards are in the front line of climate change-induced threats, as they have to cope with higher and more extreme environmental temperatures that last for longer periods [33,34]. Thus, they may have to cope with temperatures that reach or even surpass their upper thermal limits [35]. In order to survive, lizards will eventually have to modify accordingly their behavioral and physiological responses, adapting their thermophysiological plasticity [36,37]. However, when it comes to populations that have already achieved their peak of thermoregulatory effectiveness, it is questionable whether they can behaviorally buffer the continuous and sharp rise of environmental temperatures due to global warming [38,39].

In this study, we focus on such populations living in extreme environments to sketch out the thermal quality and the thermal potential of the system. We worked on two remote islets in the Aegean Sea (Greece) that are exposed to high winds and host thriving populations of the Milos wall lizard (*Podarcis milensis*). The main island populations are known to achieve high thermoregulatory effectiveness [40]. We aim to shed light on the adaptations that animals attain on small islets. We presumed that the islet populations will deviate from the main island thermoregulatory pattern and, in response to the harsh environmental conditions, will achieve higher effectiveness in thermoregulation. Using the classical approach for saurian thermoregulation [41], we seek to understand how micro-islanders may shift their thermal biology in order to survive under the challenging islet conditions. We also aim to evaluate whether there is “extra room” for the islet lizards to adapt their thermal niches and thus compensate for climate change consequences that push physiological limits.

## 2. Materials and Methods

### 2.1. Study System

The Milos wall lizard (*Podarcis milensis*) is endemic to the Milos archipelago at the Aegean Sea, Greece [42]. It is a small, robust, deep-headed lizard (Snout Vent Length—SVL—for adults: 42–70 mm) that remains active throughout the year [43]. It feeds mainly on insects and lays up to three clutches per year [44,45]. The species is listed in the IUCN Red List of Threatened Species as Vulnerable under criterion D2 due to the very small area of occupancy and its high vulnerability to stochastic events [46]. It is also listed on Annex II of the Bern Convention and Annex IV of the EU Habitats Directive 92/43.

**Figure 1:**
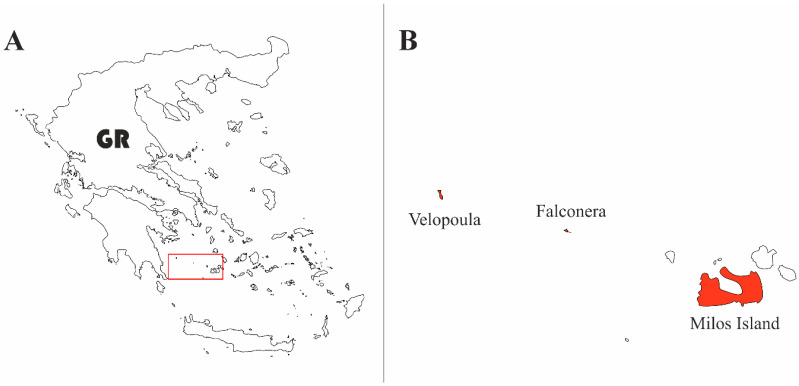
(**A**) Milos Archipelago, home of the endemic *Podarcis milensis*; the red rectangle encloses the distribution of the species; (**B**) the sampling sites are marked in red.

**Figure 2:**
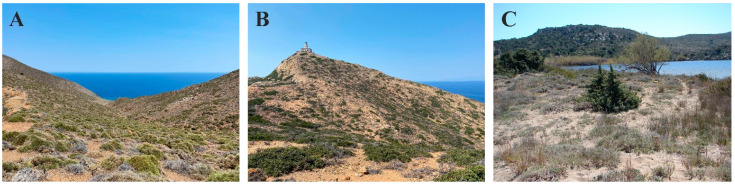
Characteristics view of the biotopes in (**A**) Falconera Islet, (**B**) Velopoula Islet, (**C**) Achivadolimni at Milos Island.

The two islets are located west of Milos and belong to the Natura 2000 network of Protected Areas (GR3000011 under the Bird Directive and GR4210011 under the Habitat Directive) and also to the new National Park of south Aegean Sea (Figure 1). The surface on both islets is uneven with steep cliffs and abrupt slopes. Falconera (1.3 km^2^, highest point 189 m) is located at a distance of 53 km from Milos (36°50′33.50″ N 23°53′13″ E) (Figure 2A). The islet owes its name to a thriving colony of Eleonora’s falcon (*Falco eleonorae*—from the Italian *falco nera* meaning “black falcon”). Velopoula (1.85 km^2^, highest point 214 m) is further west from Milos (92 km) (36°55′10.60″ N 23°27′37″ E) (Figure 2B). The predominant vegetation on both islets is phrygana, mostly *Sarcopoterium spinosum* and *Thymus capitatus*, while substrate was rocky. On Velopoula, however, we also recorded maquis plants, with *Pistacia lentiscus* standing out, that grows in a significant part of the island providing higher and denser vegetation cover. In both cases, we placed the copper models in the center of the islets, away from the spray zone, in sites with around 50% vegetation cover.

Milos Island (160.1 km^2^, highest point 751 m) belongs to the western Cyclades cluster (36°41′ N 24°25′ E). A volcanic island hosts numerous endemic species including the Milos viper (*Macrovipera schweizeri*) and *P. milensis*. The study site at Milos Island is located at the lake Achivadolimni (“the lake of clams”) and is a sandy/rocky ecosystem (Figure 2C). The dominant plants are the cade juniper (*Juniperus oxycedrus*) and conehead thyme (*Thymus capitatus*). The site where copper models were placed had also 50% vegetation cover.

### 2.2. Thermal Measurements

A total of 62 *P. milensis* adult male lizards were captured by noose in July 2023 from Velopoula and Falconera islets as well as from Milos Island (Table 1). Body temperatures were precisely measured to the nearest 0.1 °C using a quick-reading cloacal thermometer (T-4000, Miller & Weber, Inc., Queens, NY, USA). SVL (in mm) and body mass (in g) were measured with a digital caliper (Silverline 380244, accurate to 0.01 mm) and a digital scale (i500 Backlit Display, My Weight, accurate to 0.1 g), respectively. To minimize thermal shifts due to handling, the temperature of each lizard was measured within 10 s of capture. Captured lizards were transferred to the Animal Facilities at the National and Kapodistrian University of Athens for a period of two months to measure *T_pref_*. All procedures adhered to Greek National Law (Presidential Decree 67/81).

In the laboratory, lizards were housed individually in vitreous terraria (25 × 35 × 15 cm) with a sandy substrate, artificial shelters, and controlled environmental conditions (30 °C temperature, 12 h light:12 h dark photoperiod). Additional incandescent lamps (60 W) enabled behavioral thermoregulation for 8 h per day. Lizards were fed every other day with mealworms (*Tenebrio molitor*) coated with a supplement powder (TerraVit Powder, JBL GmbH & Co. KG, Neuhofen, Germany) and had unlimited access to water.

In the field, we used 28 hollow electroformed copper models that mimic the size, shape, and color of *P. milensis*, which were connected to seven data loggers (HOBO U12 4-Channel External Data Logger-U12-008) [35]. The models were placed randomly in the main types of microhabitats available to lizards (depending in exposure to sunlight and substrate), while their number of models in each type was determined based on the availability of these microhabitats (personal observations in the field)

We recorded *T_e_*s for a day during lizards’ activity time from 8:45 to 18:30 local time (UTC + 2) at 15-min intervals (*N* = 1120 records in total).

### 2.3. Lab Measurements (Preferred and Set-Point Temperatures; T_pref_ and T_set_)

To estimate the set-point temperature ranges (*T_set_*), we used the individual interquartile range (middle 50%) of the preferred body temperatures (*T_pref_*), following the approach proposed by Hertz et al. [41]. The preferred body temperatures of lizards were measured in artificial thermal arenas (100 × 25 × 25 cm). Within these arenas, a thermal gradient (from 15 to 60 °C) was created using two heating lamps (100 W and 60 W) at one end and two ice bags at the opposite wall as determined by hollow copper models of a lizard [48]. Before temperature recordings, lizards were allowed to acclimate for an hour [49,50]. *T_pref_*s were recorded every hour over a five-hour period (from 10:00 am to 3:00 pm) using a cloacal Miller–Weber thermometer.

### 2.4. Effectiveness of Thermoregulation (E)

We calculated the effectiveness of thermoregulation as the ability of an animal to maintain its body temperature closer to their *T_pref_* rather than *T_e_*. Two indices were used: the one proposed by Hertz et al. [41]: *Ε* = 1 − (${\bar{d}}_{b}/{\bar{d}}_{e}$) and the alternative by Blouin–Demers and Weatherhead [47] (${\bar{d}}_{e}-{\bar{d}}_{b}$) due to innate biases of the former to estimate similar *E* values for different ${\bar{d}}_{b}$ and ${\bar{d}}_{e}$ combinations. The mean *d*_b_ here (${\bar{d}}_{b}$) is estimated as the mean absolute deviation of *T_b_* from *T_set_* and thus defines the accuracy of thermoregulation. The thermal quality of a given habitat is an index defined by ${\bar{d}}_{e}$ and is estimated as the mean absolute deviation of *T_e_* from *T_set_*. Thermoregulatory effectiveness describes the physiological and/or behavioral processes that animals employ to regulate their body temperature to efficient levels.

### 2.5. Statistical Analysis

The normality of the data was assessed using the Kolmogorov–Smirnov and Lilliefors test. When parametric assumptions were not satisfied, nonparametric tests were employed; otherwise, parametric tests were used. Permutation ANOVAs with 1000 iterations were conducted to test for differences between habitats on field body temperatures (*T_b_*) and preferred body temperatures (*T_pref_*). Post hoc tests, including Games Howell, were employed to identify significant differences between pairs of group means. Permutation ANOVA was carried out to compare the operative temperatures (*T*_e_) between sites. Generalized linear models (GLMs) fitting a zero-inflated gamma distribution were employed to assess the differences in thermal habitat quality (${\bar{d}}_{e}$) and thermoregulation accuracy (${\bar{d}}_{b}$). Finally, the bootstrap resampling (repeated 1000 times) method was carried out to generate the 95% confidence intervals for the two indices of effectiveness of thermoregulation (*E* and $\bar{d_{e}}$-$\bar{d_{b}}$).

## 3. Results

### 3.1. Body Temperatures (T_b_ and T_pref_)

In Table 1, we summarized the values for thermal parameters ruling the three populations of *P. milensis* thermoregulation. The comparison of Tbs between populations showed significant differences (F_2, 59_ = 9.30, *p* < 0.001) with Falconera lizards achieving significant higher body temperatures in the field than Milos and Velopoula conspecifics (Games Howell pairwise test, Falconera–Velopoula: *p* = 0.049 Milos–Velopoula: *p* = 0.804 and Milos–Falkonera *p* = 0.004). Likewise, we found that Falconera lizards select for higher body temperatures (*Tpref*; 33.14 ± 0.46 °C) than Milos (32.03 ± 0.84 °C) and Velopoula (32.24 ± 1.19 °C) ones (F_2, 39_ = 6.09, *p* = 0.005) (Games Howell pairwise test, Falconera–Velopoula: *p* = 0.048 Milos–Velopoula: *p* = 0.649 and Milos–Falkonera *p* = 0.024).

### 3.2. Operative Temperatures (T_e_)

The mean T_e_ values exhibited a notable decrease on Milos Island (i.e., Achivadolimni) followed by the Falconera and Velopoula islets (permANOVA; based on 9999 iterations F_2, 3217_ = 375.34, *p* < 0.001). The detected differences in T_e_ between populations resulted from the higher frequency of lower temperatures recorded on the island of Milos (T_e_ ranged from 19.4 to 52.3 °C) compared to the two islets (T_e_s on average ranged from 25 to 64.5 °C), which is probably an indicator of the lower vegetation coverage and the less shady habitats of the islets (Table 1).

### 3.3. Effectiveness of Thermoregulation

The mean deviation of T_e_ from T_set_ (${\bar{d}}_{e}$; low values show a habitat of higher thermal quality, where the majority of recorded T_e_ values fall within the T_set_ limits) showed significant differences across populations (all pairwise Ps < 0.001; GLM), with Velopoula islet showing the poorer thermal quality, which was followed by Falconera and Milos (Table 1). On the islets, the majority of T_e_s were above T_set_ (on average 81.5%) during the day, whereas on Milos Island, the distribution of T_e_s around T_set_ was more equally distributed (55% were higher and 28% were lower than the maximum and minimum T_set_, respectively).

Likewise, the accuracy of thermoregulation (${\bar{d}}_{b}$) was significantly different among the three sites (*p* = 0.016) with Falconera lizards achieving the lowest accuracy of thermoregulation (Table 1). No further differences were observed between Velopoula and Milos lizards. Notably, 40–73% of the recorded T_b_s for the three populations were between the minimum and maximum T_set_, supporting this high accuracy of thermoregulation that was observed for *P. milensis* (d_b_ ranged from 0.22 to 0.56).

Finally, the effectiveness of thermoregulation showed significant differences among populations (Table 1). Although all three populations thermoregulate with high efficiency (Table 1), Velopoula and Falconera lizards exhibited among the highest effectiveness that have been recorded on *Podarcis* lizards (0.95 and 0.97, respectively). Bootstrap resampling correcting for pairwise comparisons highlighted those differences, revealing a similar pattern of thermoregulatory effectiveness for the two islets than the main island of Milos (E bootstrap values were 972 and 1000 times higher than Milos on Velopoula and Falconera sites, respectively). Interestingly, the index of Blouin–Demers and Weatherhead (2001) (${\bar{d}}_{e}-{\bar{d}}_{b}$) yielded higher thermoregulation values for Velopoula (11.54) and Falconera (6.97) lizards and lower in the case of the Milos population (4.27) (Table 1).

## 4. Discussion

Typically, islands have milder climate and higher thermal quality than the mainland thanks to the buffering effect of the surrounding sea [10,11,19]. Nonetheless, small islets deviate from this pattern due to significant differences in size, altitude, habitat homogeneity and vegetation cover [32,51]. The demanding nature of small islets that are exposed to high winds and provide a limited variety of microhabitats and shelters was reflected in our findings. Lizards from the focal islets differed in all thermal parameters in response to the challenging micro-insular conditions. This was clear in the effectiveness of thermoregulation that was higher in the two islets compared to the Milos population.

**Figure 3:**
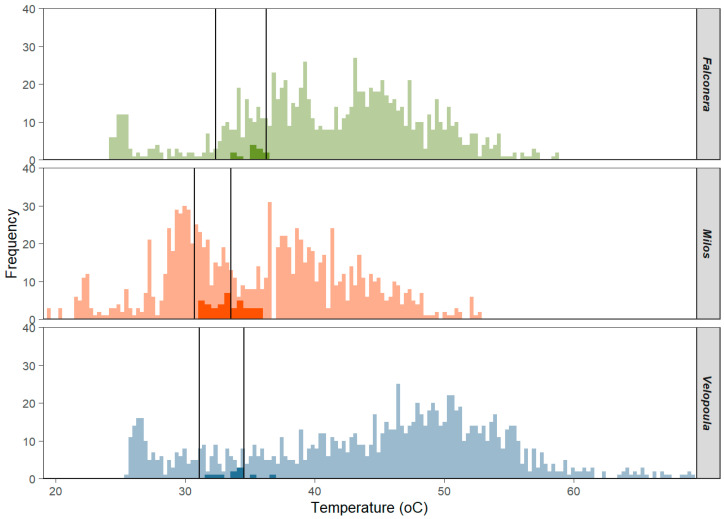
Distribution of the mean active body temperature in the field (*T_b_*; dark color) and the mean operative environmental temperature (*T_e_*; open color) in *Podarcis milensis* for each population. Black vertical lines indicate the set-point temperature range (*T_set_*) measured under laboratory conditions.

**Figure 4:**
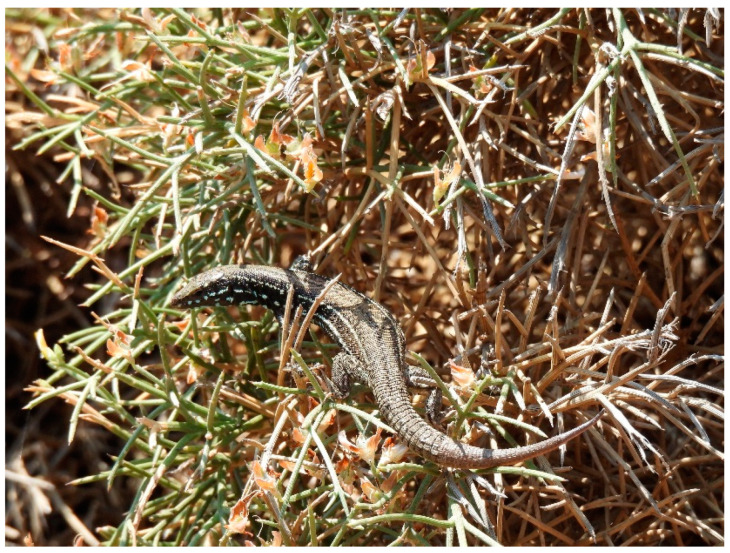
An adult *Podarcis milensis* female on Falconera Islet while basking on a branch of a bush, sheltered by high winds.

In line with our working hypothesis, the comparison between the three populations revealed significant differences in body temperatures (Table 1). Both *T_b_* and *T_pref_*, the body temperatures that lizards achieved in the field and in the lab, respectively, were higher in the two islets with the Falconera population receiving the higher values and Velopoula following (Table 1, Figure 3). At first glance, high body temperatures in such environments seem paradoxical. The increased wind speed on the islets should imply lower *T_b_* and favor the selection of lower *T_pref_* in the lab, e.g., [29,32,52]. However, the distinct characteristics of the islets may play a role in affecting body temperatures. For instance, the higher vegetation allows the lizards to bask through the branches of the bushes, thus avoiding full exposure to strong winds, which is a behavior that we observed in the field (Figure 4). Furthermore, high winds may have the opposite effect: lizards may reach higher *T_b_* and *T_pref_* in the anticipation of evaporating cooling [53]. On the other hand, physiological and/or behavioral strategies may account for this outcome. Lizards are known to shuttle between favorable microhabitats and thus buffering the effect of climate conditions [46].

Operative temperatures also differed between the islands the higher the values recorded on the two islets (Table 1, Figure 3). The difference in the islet *T_e_s* was depicted in the higher values of *d_e_* and the mean deviation of *T_e_* from *T_pref_* (Table 1). The mean *d_e_* is a measure of the habitat thermal quality [41]. The higher values for Velopoula (12.10) and Falconera (7.19) sketch out much more challenging, in terms of temperature, habitats than Milos (4.76). Lizards in low thermal quality habitats, expressed as high *d_e_*, are expected to achieve effective thermoregulation [36,54]. In other words, the focal islets lizards have to effectively thermoregulate in order to survive in these thermally harsh habitats.

**Figure 5:**
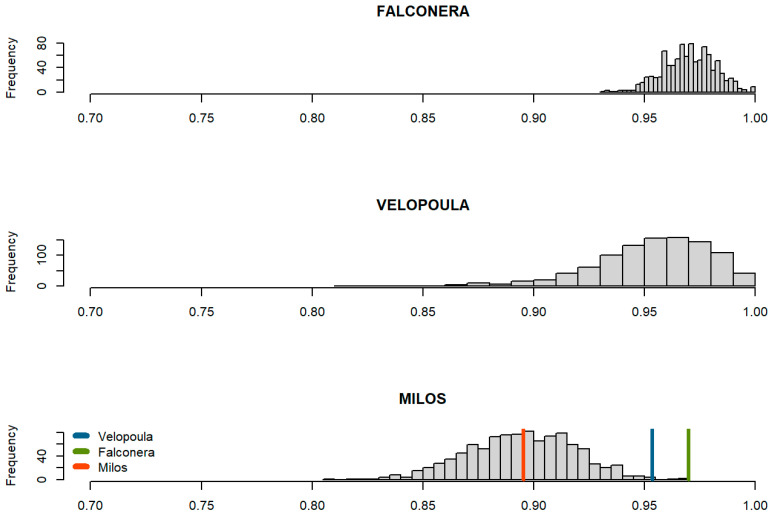
Distribution plot of *E* bootstrapped values obtained from Hertz et al. [41] index, showing the overlaps among *Podarcis milensis* populations.

Indeed, the effectiveness of thermoregulation reached its higher values on the two islets (Table 1, Figure 5). This deviation clearly results from the application of the classical formula [41] (Figure 5). A similar E value was calculated in a previous work [40], though it was somehow higher (0.95 vs. 0.89). This discrepancy should be attributed to methodological reasons (we recorded *T_e_s* every 15 min for 9 h during July, while in the 2005 study, *T_e_s* were recorded hourly for 4 h in August). We also used the approach proposed by Blouin–Demers and Weatherhead [47] to unveil the “hidden” biases of the classical formula in which the same *E* values were obtained when different combinations of mean *d_e_*s and *d_b_*s are considered. This approach uses the difference between mean db and mean de. The magnitude of the difference is a measure of how much an animal departs from thermoconformity, and it serves as an index of thermoregulatory effectiveness. This supplementary approach strikingly highlighted the differences in *E* values: Velopoula lizards thermoregulate more effectively (almost threefold compared to Milos), which is followed by Falconera ones and, last, by the lizards from Milos (Table 1). The large-scale differences between mean db and de that were masked by the classical formula in the case of Velopoula were revealed when the complementary approach was used.

Climate and geomorphological parameters do differ between Milos and the two islets. Elevation is considered as an index of insular environmental heterogeneity since islands with a strong mountain profile are characterized by higher habitat heterogeneity when compared to islands with low mountains [27]. Velopoula and Falconera are quite exposed to strong winds (average wind speed during July: 22 km/h; meteoblue.com, accessed on 22 August 2024). Because of their low elevation (290 m and 189 m, respectively), they provide limited protection. This fact is verified by the impressive fluctuations of the islet *T_e_s* (Figure 3) that receive extremely high values (Falconera: 58.9 °C and Velopoula: 69.4 °C—Table 1). In addition, the focal habitat on Milos was characterized by a larger microhabitat heterogeneity. A spacious belt zone of land between lake and sea, with sand dunes, rocks, stones and vegetation varying from low scrubs to trees [40], certainly provides a wider variety of microhabitats among which lizards may shuttle. Moreover, the distribution of the operative temperatures on the two islets was skewed toward the upper thermal limit, and for the longest period, *T*_e_s values were higher than *T_pref_* compared to Milos (Figure 3).

Lizards from low-quality habitats thermoregulate effectively to survive [32,55], and this is what the islet lizards do in our study system. Our findings comply with those from other studies on *Podarcis* lizards living on similar Mediterranean habitats [31,32], as shown in Table 2 in [56]: islet *Podarcis* achieve high *E* values in order to cope with their demanding habitats. They reach high *E* values, their body temperatures show limited diel variation (an index of thermoregulatory precision) and achieve low *d_b_* (a benchmark for thermoregulatory accuracy) (Figure 3). In sum, the lizards from Velopoula and Falconera cope well with their demanding habitat and perform a precise, accurate and effective thermoregulation. But, could they deal with a further deterioration of their low-quality habitat? Possible alternatives, such as avoiding the hot mid-day hours and being active earlier in the morning when temperatures are still tolerable, should be further examined.

The Aegean Sea, just between Europe and Africa, faces a great risk because of global warming [57]. Over the last 40 years, a steady trend of increasing average temperature has been recorded in the Aegean region [58]. A further rise in the temperature of the remote and small Aegean islets would directly impact the lizards that live in these already thermally challenging habitats. The saurian thermal physiology is largely affected by environmental temperatures [14,15]. Therefore, populations that have already achieved their peak of thermoregulation and inhabit extreme habitats such as mountaintops or remote islets have—literally—nowhere to go [59,60]. Global warming is already preventing lizards to buffer environmental change through behavioral shifts [39,61]. Continued annual surveys of such populations over the few coming years will shed light on their physiological resilience to climate change and provide insights on their conservation. At the same time, conservation actions should not be neglected. Microclimate management through mild human activities that have been used for millennia in the Aegean insular landscape (e.g., drystone walls and terraces) and can create favorable microclimates [62] may prove to be invaluable mitigation strategies.

## 5. Conclusions

The Falconera and Velopoula populations achieve highly effective thermoregulation in response to the demanding conditions prevailing on the islets. Interestingly, they selected for higher body temperatures, both in the field and the lab, despite the strong winds that blowing in the area. This could be attributed to physiological adjustments [53] or to idiosyncratic features of each islet [31,32]. Although the islet populations have reached the maximum of their thermoregulatory effectiveness, especially during summer when the thermal quality is usually lower in the Mediterranean Basin [63], this may prove to be too little to offset the effects of global warming.

## Figures and Tables

**Table 1:** Values for field body (*T_b_*), preferred body (*T_pref_*) and operative (*T_e_*) temperatures in the three different sites. Means ± standard deviation; range and sample size (*N*). The indices of effectiveness of thermoregulation. *E_H_* refers to the equation of Hertz et al. [41], whereas *E_B_* to Blouin–Demers and Weatherhead [47].

Population	*T_b_* (°C)	*T_pref_* (°C)	*T_e_* (°C)	*d_b_* (°C)	*d_e_* (°C)	*E_H_*	*E_B_*
Falconera islet	35.23 ± 0.85	33.14 ± 1.19	41.25 ± 7.27	0.22 ± 0.28	7.19 ± 5.40	0.97	6.97
	(33.80–36.20)	(31.66–35.17)	(24.40–58.90)	(0.0–0.68)	(0.0–23.38)		
	*N* = 12	*N* = 13	*N* = 28	*N* = 12	*N* = 28		
Velopoula islet	34.00 ± 1.52	32.24 ± 0.46	44.84 ± 9.59	0.56 ± 0.95	12.10 ± 7.78	0.95	11.54
	(31.70–37.00)	(31.59–33.25)	(25.60–69.40)	(0.0–3.04)	(0.0–35.44)		
	*N* = 10	*N* = 11	*N* = 28	*N* = 10	*N* = 28		
Milos Island	33.38 ± 1.37	32.03 ± 0.84	35.68 ± 6.67	0.49 ± 0.74	4.76 ± 4.38	0.89	4.27
	(31.20–35.90)	(30.23–33.80)	(19.40–52.90)	(0.0–2.37)	(0.0–19.37)		
	*N* = 40	*N* = 18	*N* = 28	*N* = 40	*N* = 28		

## Data Availability

The data presented in this study are available on request from the corresponding author.
